# Comprehensive epithelial tubo-ovarian cancer risk prediction model incorporating genetic and epidemiological risk factors

**DOI:** 10.1136/jmedgenet-2021-107904

**Published:** 2021-11-29

**Authors:** Andrew Lee, Xin Yang, Jonathan Tyrer, Aleksandra Gentry-Maharaj, Andy Ryan, Nasim Mavaddat, Alex P Cunningham, Tim Carver, Stephanie Archer, Goska Leslie, Jatinder Kalsi, Faiza Gaba, Ranjit Manchanda, Simon Gayther, Susan J Ramus, Fiona M Walter, Marc Tischkowitz, Ian Jacobs, Usha Menon, Douglas F Easton, Paul Pharoah, Antonis C Antoniou

**Affiliations:** 1 Centre for Cancer Genetic Epidemiology, Department of Public Health and Primary Care, University of Cambridge, Cambridge, UK; 2 Centre for Cancer Genetic Epidemiology, Department of Oncology, University of Cambridge, Cambridge, UK; 3 MRC Clinical Trials Unit, Institute of Clinical Trials & Methodology, University College London, London, UK; 4 The Primary Care Unit, Department of Public Health and Primary Care, University of Cambridge, Cambridge, UK; 5 Department of Women’s Cancer, University College London Institute for Women's Health, London, UK; 6 Department of Epidemiology and Public Health, University College London Research, London, UK; 7 CRUK Barts Cancer Centre, Wolfson Institute of Preventive Medicine, London, UK; 8 Department of Gynaecological Oncology, Barts Health NHS Trust, London, UK; 9 Department of Health Services Research, London School of Hygiene & Tropical Medicine, London, UK; 10 Center for Bioinformatics and Functional Genomics, Cedars-Sinai Medical Center Samuel Oschin Comprehensive Cancer Institute, Los Angeles, California, USA; 11 University of New South Wales, School of Women's and Children's Health, Randwick, New South Wales, Australia; 12 Adult Cancer Program, Lowy Cancer Research Centre, University of New South Wales, Sydney, New South Wales, Australia; 13 Department of Medical Genetics, NIHR Cambridge Biomedical Research Centre, University of Cambridge, Cambridge, UK

**Keywords:** genetics, public health, early diagnosis, clinical decision-making, genetic counseling

## Abstract

**Background:**

Epithelial tubo-ovarian cancer (EOC) has high mortality partly due to late diagnosis. Prevention is available but may be associated with adverse effects. A multifactorial risk model based on known genetic and epidemiological risk factors (RFs) for EOC can help identify women at higher risk who could benefit from targeted screening and prevention.

**Methods:**

We developed a multifactorial EOC risk model for women of European ancestry incorporating the effects of pathogenic variants (PVs) in *BRCA1*, *BRCA2*, *RAD51C*, *RAD51D* and *BRIP1*, a Polygenic Risk Score (PRS) of arbitrary size, the effects of RFs and explicit family history (FH) using a synthetic model approach. The PRS, PV and RFs were assumed to act multiplicatively.

**Results:**

Based on a currently available PRS for EOC that explains 5% of the EOC polygenic variance, the estimated lifetime risks under the multifactorial model in the general population vary from 0.5% to 4.6% for the first to 99th percentiles of the EOC risk distribution. The corresponding range for women with an affected first-degree relative is 1.9%–10.3%. Based on the combined risk distribution, 33% of *RAD51D* PV carriers are expected to have a lifetime EOC risk of less than 10%. RFs provided the widest distribution, followed by the PRS. In an independent partial model validation, absolute and relative 5-year risks were well calibrated in quintiles of predicted risk.

**Conclusion:**

This multifactorial risk model can facilitate stratification, in particular among women with FH of cancer and/or moderate-risk and high-risk PVs. The model is available via the CanRisk Tool (www.canrisk.org).

## Introduction

Epithelial tubo-ovarian cancer (EOC), the seventh most common cancer in women globally, is often diagnosed at late stage and is associated with high mortality. There were 7443 new cases of EOC and 4116 deaths from EOC annually in the UK in 2015–2017.^1^ Early detection could lead to an early-stage diagnosis, enabling curative treatment and reducing mortality. Annual multimodal screening using a longitudinal serum CA125 algorithm in women from the general population resulted in significantly more women diagnosed with early-stage disease but without a significant reduction in mortality.^2^ Four-monthly screening using the same multimodal approach also resulted in a stage shift in women at high risk (>10% lifetime risk of EOC).^3^ Currently, risk-reducing bilateral salpingo-oophorectomy (RRSO), on completion of their families, remains the most effective prevention option,^4^ and it has been recently suggested that RRSO would be cost-effective in postmenopausal women at >4% lifetime EOC risk.^5 6^ Beyond surgical risk, bilateral oophorectomy may be associated with increased cardiovascular mortality^7^ and a potential increased risk of other morbidities such as parkinsonism, dementia, cardiovascular disease and osteoporosis,^8 9^ particularly in those who do not take menopausal hormone therapy (MHT).^10^ Therefore, it is important to target such prevention approaches to those at increased risk who are most likely to benefit.

Over the last decade, there have been significant advances in our understanding of susceptibility to EOC. After age, family history (FH) is the most important risk factor (RF) for the disease. Approximately 35% of the observed familial relative risk (FRR) can be explained by rare pathogenic variants (PVs) in the *BRCA1*, *BRCA2, RAD51C*, *RAD51D* and *BRIP1* genes.^11–14^ Recent evidence suggests that *PALB2*, *ATM*, *MLH1*, *MSH2* and *MSH6* are also involved in the EOC genetic susceptibility.^14–18^ Common variants, each of small effect, identified through genome-wide association studies,^19 20^ explain a further 4%. Several epidemiological RFs are also known to be associated with EOC risk, including use of MHT, Body Mass Index (BMI), history of endometriosis, use of oral contraception, tubal ligation and parity.^21–26^ Despite these advances, those at high risk of developing EOC are currently identified mainly through FH of the disease or on the basis of having PVs in *BRCA1* and *BRCA2*. However, more personalised risk prediction could be achieved by combining data on all known epidemiological and genetic RFs. The published EOC prediction models consider either RFs^24 25 27^ or common variants.^24 28^ No published EOC risk prediction model takes into account the simultaneous effects of the established EOC susceptibility genetic variants (rare and common), residual FH and other known RFs.

Using complex segregation analysis, we previously developed an EOC risk prediction algorithm that considered the effects of PVs in *BRCA1* and *BRCA2* and explicit FH of EOC and breast cancer (BC).^11^ The algorithm modelled the residual, unexplained familial aggregation using a polygenic model that captured other unobserved genetic effects. The model did not explicitly include the effects of other established intermediate-risk PVs in genes such as *RAD51C*, *RAD51D* and *BRIP1,* 12–14 29 which are now included on routine gene panel tests, the effects of recently developed EOC Polygenic Risk Scores (PRSs) or the known RFs.

Here we present a methodological framework for extending this model to incorporate the explicit effects of PVs in *RAD51C*, *RAD51D* and *BRIP1* for which reliable age-specific EOC risk estimates are currently available, up-to-date PRSs and the known EOC RFs (table 1). We used this multifactorial model to evaluate the impact of negative predictive testing in families with rare PVs and to assess the extent of EOC risk stratification that can be achieved in the general population, women with a FH of EOC and those carrying rare PVs. We evaluated the performance of a subset of this model in the UK Collaborative Trial of Ovarian Cancer Screening (UKCTOCS),^2^ where women from the general population were followed up prospectively.

**Table 1:** Summary of components of the EOC risk model

RF group	RF category	Comments
FH	Explicit FH of ovarian and other cancers (breast, prostate, male breast and pancreatic)	Considers families of arbitrary size and structure, including affected and unaffected relatives
Sex		Sex of all family members
Age		Ages at cancer diagnosis or current ages/age at death of family members
Genetic factors		
Rare truncating/pathogenic variants	*BRCA1*	
	*BRCA2*	
	*RAD51D*	
	*RAD51C*	
	*BRIP1*	
Common genetic variants	Polygenic Risk Score	Explaining 5% of the polygenic variance
Unobserved genetic effects	Residual polygenic component	Accounts for the residual familial aggregation of EOC
Lifestyle/hormonal/reproductive		
	Height	Measured in cm (five categories)
	Body Mass Index	Measured in kg/m^2^ (three categories)
	Parity	Number of live births (three categories)
	Endometriosis	Yes/no
	Use of oral contraception	Years of use (five categories)
	Use of hormone replacement therapy	Never/ever
	Tubal ligation	Yes/no
Breast tumour pathology	Oestrogen, progesterone, HER2 receptor, CK14, CK5/6 status	As implemented in the BOADICEA breast cancer model
Demographic factors		
Country of origin	Country	Defines the underlying incidences used
Birth cohort	Defined by the person’s year of birth	Eight calendar year-specific sets of incidences
Family ethnicity	Ashkenazi Jewish origin	

BOADICEA, Breast and Ovarian Analysis of Disease Incidence and Carrier Estimation Algorithm; EOC, epithelial tubo-ovarian cancer; FH, family history; RF, risk factor.

## Methods

### EOC risk prediction model development

No large datasets are currently available that include data on all known genetic and other EOC RFs. Therefore, we used a synthetic approach, described previously,^30^ to extend our previous EOC model^11^ by capitalising on published estimates of the associations of each RF with EOC. This approach was shown to provide valid risk estimates in the case of BC.^30–32^

Under the assumption that the effects of rare PVs, RFs and polygenic component are multiplicative on EOC risk, the incidence at age t for individual *i* was modelled as

(1) $$\lambda^{\left(i\right)}\left(t\right)=\lambda_{0}\left(t\right)exp\left(\beta_{MG}^{\left(i\right)}\left(t\right)+\sum_{\rho }\beta_{RF\rho }^{\left(i\right)}\left(t\right)\cdot z_{RF\rho }^{\left(i\right)}+\beta_{PG}\left(t\right)x_{P}^{\left(i\right)}\right),$$

where $\lambda_{0}(t)$ is the baseline incidence. $\beta_{MG}^{\left(i\right)}(t)$ is the age-specific log-relative risk (log-RR) associated with individual i ’s PV carrier status (explained further), relative to the baseline. The log-RR for non-carriers is 0. $x_{P}^{\left(i\right)}$ is the polygenotype for individual i , assumed to follow a standard normal distribution in the general population, and $\beta_{PG}(t)$ is the age-specific log-RR associated with the polygene, relative to the baseline incidence. $\beta_{RF\rho }^{\left(i\right)}(t)$ is the log-RR associated with risk-factor ρ at age t , which may depend on PV carrier status, and $z_{RF\rho }^{\left(i\right)}$ is the corresponding indicator variable showing the category of risk-factor ρ for the individual. The baseline incidence was determined by constraining the overall incidences to agree with the population EOC incidence. To allow appropriately for missing RF information, only those RFs measured on a given individual are considered.

#### Major gene (MG) effects

To include the effects of *RAD51D*, *RAD51C* and *BRIP1*, we used the approach described previously where PVs in these genes were assumed to be risk alleles of a single MG locus.^33^ A dominant model of inheritance was assumed for all rare PVs. To define the penetrance, we assumed the following order of dominance when an individual carried more than one PV (ie, the risk was determined by the highest-risk PV and any lower-risk PVs ignored): *BRCA1*, *BRCA2*, *RAD51D*, *RAD51C* and *BRIP1*.^33^ The population allele frequencies for *RAD51D*, *RAD51C* and *BRIP1* and EOC relative risks (RRs) were obtained from published data (online supplemental table S3).^14 29^ Although PVs in *PALB2*, *ATM*, *MLH1*, *MSH2* and *MSH6* have been reported to be associated with EOC risk, PVs in *MLH1*, *MHS2* and *MSH6* are primarily associated with risk of specific subtypes of EOC (endometrioid and clear cell),^17^ and at the time of development, precise EOC age-specific risk estimates for *PALB2* and *ATM* PV carriers were not available. Therefore, these were not considered at this stage.

10.1136/jmedgenet-2021-107904.supp1 Supplementary data

#### Epidemiological RFs

The RFs incorporated into the model include parity, use of oral contraception and MHT, endometriosis, tubal ligation, BMI and height. We assumed that the RFs were categorical and that individuals’ categories were fixed for their lifetime, although the RRs were allowed to vary with age. The RR estimates used in equation (1) and population distributions for each RF were obtained from large-scale external studies and from national surveillance data sources using a synthetic approach as previously described.^30^ Where possible, we used RR estimates that were adjusted for the other RFs included in the model and distributions from the UK. Details of the population distributions and RRs used in the model are given in online supplemental table S2. As in the Breast and Ovarian Analysis of Disease Incidence and Carrier Estimation Algorithm (BOADICEA),^30^ in order to decrease the runtime, we combined the RFs with age-independent RRs into a single factor (specifically parity, tubal ligation, endometriosis, BMI and height).

#### Incorporating PRSs

We included an EOC susceptibility PRS, assumed to form part of the polygene, using the methods previously developed.^11 30^ The polygenic component decomposes into a measured component due to the PRS ( $x_{PRS}$ ) and an unmeasured component representing other familial effects ( $x_{R}$ ):

(2) $$x_{P}=x_{PRS}+x_{R}.$$

$x_{PRS}$ summarises the effects of multiple common variants and is assumed normally distributed with mean 0 and variance $\alpha^{2}$ in the general population, with 0≤α≤1 . The parameter $\alpha^{2}$ is the proportion of the overall polygenic variance (after excluding the effects of all MGs) explained by the PRS. $x_{R}$ is normally distributed with mean 0 and variance $1-\alpha^{2}$ . The approach used to calculate $\alpha^{2}$ is described in the online supplemental material. This implementation allows the effect size of the PRS to be dynamically varied, allowing an arbitrary PRS.

Here, to illustrate the model’s risk-stratification potential, we considered the latest validated EOC PRS developed by the Ovarian Cancer Association Consortium,^34^ which is composed of 36 variants (online supplemental table S1) and has a log variance of 0.099, accounting for 5.0% of the overall polygenic variance in the model. This 36-variant PRS was found to perform equally well as those comprising more variants based on penalised regression or Bayesian approaches.^34^

#### Other model components

The previous version^11^ modelled the incidence of EOC and first female BC. To align with BOADICEA,^30^ the model was extended to take account of female contralateral BC and the associations of *BRCA1/2* PVs with pancreatic cancer, male BC and prostate cancer (online supplemental methods).

### Model validation

#### Study subjects

A partial model validation was carried out in a nested case–control sample of women of self-reported European ancestry participating in UKCTOCS. Based on the data available, we were able to validate the model on the basis of FH, PRS and RFs. Details of the UKCTOCS study design, blood sampling process, DNA extraction and processing, variant selection, genotyping and data processing are described in the online supplemental Methods and published elsewhere.^35^ Women with an FH of two or more relatives with EOC or who were known carriers of *BRCA1/2* PVs were not eligible to participate in UKCTOCS. In summary, the following self-reported information was collected at recruitment and used for model validation: parity, use of oral contraception and MHT, tubal ligation, BMI and height (online supplemental table S4). As the study participants were genotyped for only 15 Single Nucleotide Polymorphisms (SNPs) known at the time to be associated with EOC risk, it was not possible to use the more recently developed PRS for model validation. Instead, as the model can accommodate an arbitrary PRS, a PRS based on the 15 available SNPs was used^35^ (online supplemental table S5), for which $\alpha^{2}=0.037$ . The UKCTOCS study participants were independent of the sets used to generate this PRS.^35^ Study participants were not screened for PVs in *BRCA1*, *BRCA2*, *RAD51C*, *RAD51D* or *BRIP1*.

#### Pedigree construction

The UKCTOCS recruitment questionnaire collected only summary data on FH of BC and EOC. Since the risk algorithm uses explicit FH information, these data were used to reconstruct the pedigrees, which included information on incidences in the first-degree and second-degree relatives (online supplemental methods).

#### Statistical analysis

All UKCTOCS participants were followed up using electronic health record linkage to national cancer and death registries. For this study, they were censored at either their age at EOC, their age at other (non-EOC) first cancer diagnosis, their age at death or age 79. To assess the model performance, a weighted approach was used whereby each participant was assigned a sampling weight based on the inverse of the probability of being included in the nested case–control study, given their disease status. Since all incident cancer cases were included, cases were assigned a weight of 1. The cases were matched to two random controls (women with no EOC cancer) recruited at the same regional centre, age at randomisation and year at recruitment.

We assessed the model calibration and discrimination of the predicted 5-year risks. Women older than 74 years at entry were excluded. Cases that developed EOC beyond 5 years were treated as unaffected. For controls with a less than 5 years of follow-up, we predicted the EOC risks to the age at censoring. For all other controls and cases, we predicted 5-year risks.

To assess model calibration, we partitioned the weighted sample into quintiles of predicted risk. Within each quintile, we compared the weighted mean of predicted risk to the weighted observed incidence using the Hosmer-Lemeshow (HL) χ^2^ test.^36^ To assess RR calibration, the predicted and observed RRs were calculated relative to the corresponding means of risks over all quintiles. We also compared the expected (E) with the observed (O) EOC risk within the prediction interval by calculating the ratio of expected to observed cases (E/O). The 95% CI for the ratio was calculated assuming a Poisson distribution.^37^

We assessed the model discrimination between women who developed and did not develop EOC within 5 years using the area under the receiver operating characteristic curve (AUC) (online supplemental methods).

## Results

### Model description

**Figure 1:**
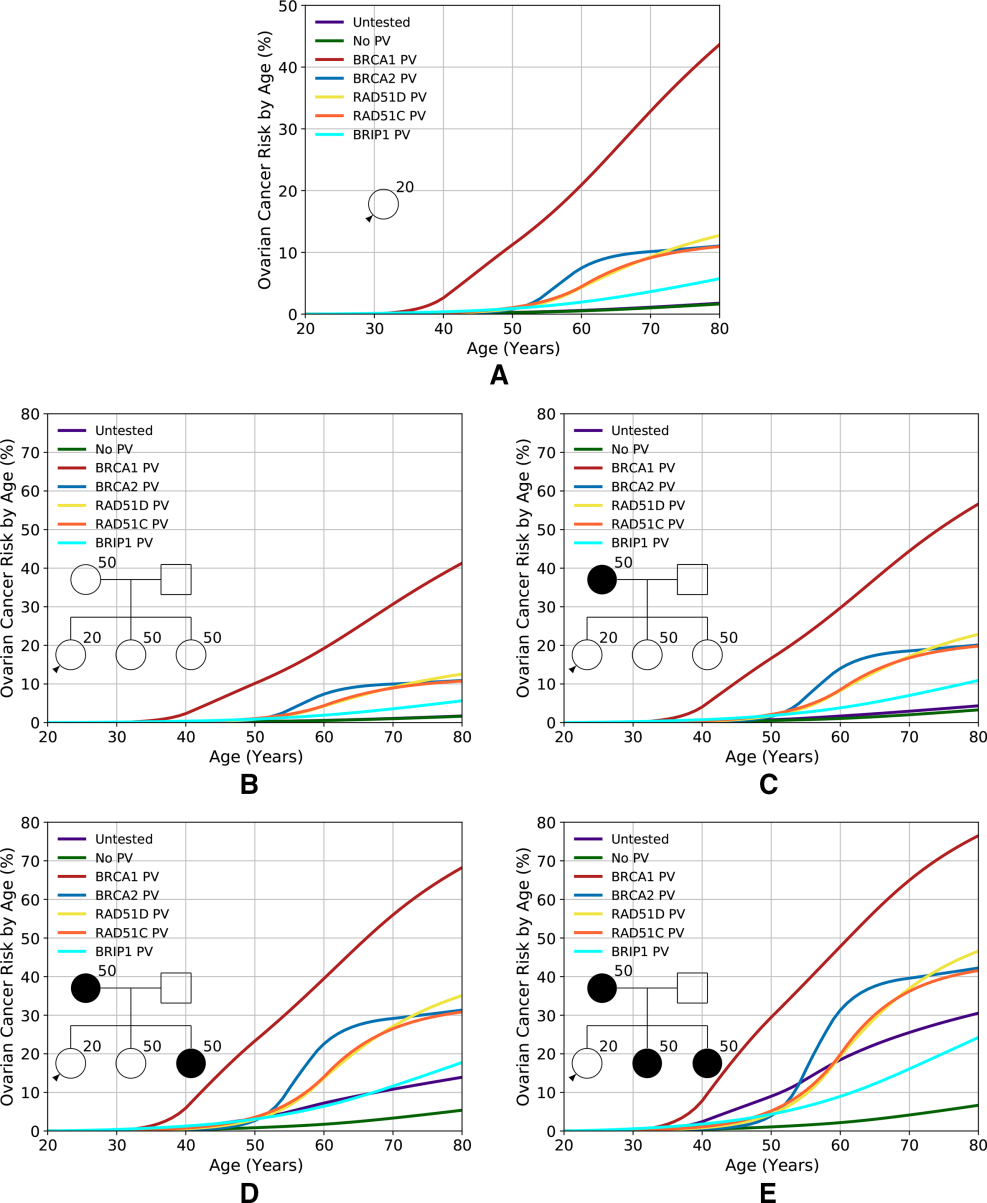
Predicted lifetime (age 20–80 years) EOC risk by PV and family history. Each fgure shows the risks assuming the woman is untested, has no PVs or carries a PV in *BRCA1, BRCA2, RAD51D, RAD51C* or *BRIP1*. (A) Assuming an unknown family history. (B–E) Assuming an increasing number of affected first-degree relatives, as indicated by the pedigree diagram inserts. Predictions are based on UK EOC population incidence. EOC, epithelial tubo-ovarian cancer; PV, pathogenic variant.

*RAD51D*, *RAD51C* and *BRIP1*, based on the assumed allele frequencies and RRs, account for 2.5% of the overall model polygenic variance. Figure 1 shows the predicted EOC risks for carriers of PVs in *BRCA1*, *BRCA2*, *RAD51D*, *RAD51C* and *BRIP1* for various FH scenarios. With unknown FH, the risks for carriers of PVs in *RAD51D*, *RAD51C* and *BRIP1* are 13%, 11% and 6%, respectively. For example, for a *BRIP1* PV carrier, the risk varies from 6% for a woman without EOC FH to 18% for a woman with two affected first-degree relatives. The model can also be used to predict risks in families in which PVs are identified but where other family members test negative (online supplemental figure S1). For women with an FH of EOC, the reduction in EOC risk after negative predictive testing is greatest for *BRCA1* PVs, with the risks being close to (though still somewhat greater than) population risk. This effect was most noticeable for women with a strong FH. Although a risk reduction is also seen for women whose mother carried a PV in *BRCA2*, *RAD51D*, *RAD51C* or *BRIP1*, the reduction is less marked. As expected, the predicted risks are still elevated compared with the population.

**Figure 2:**
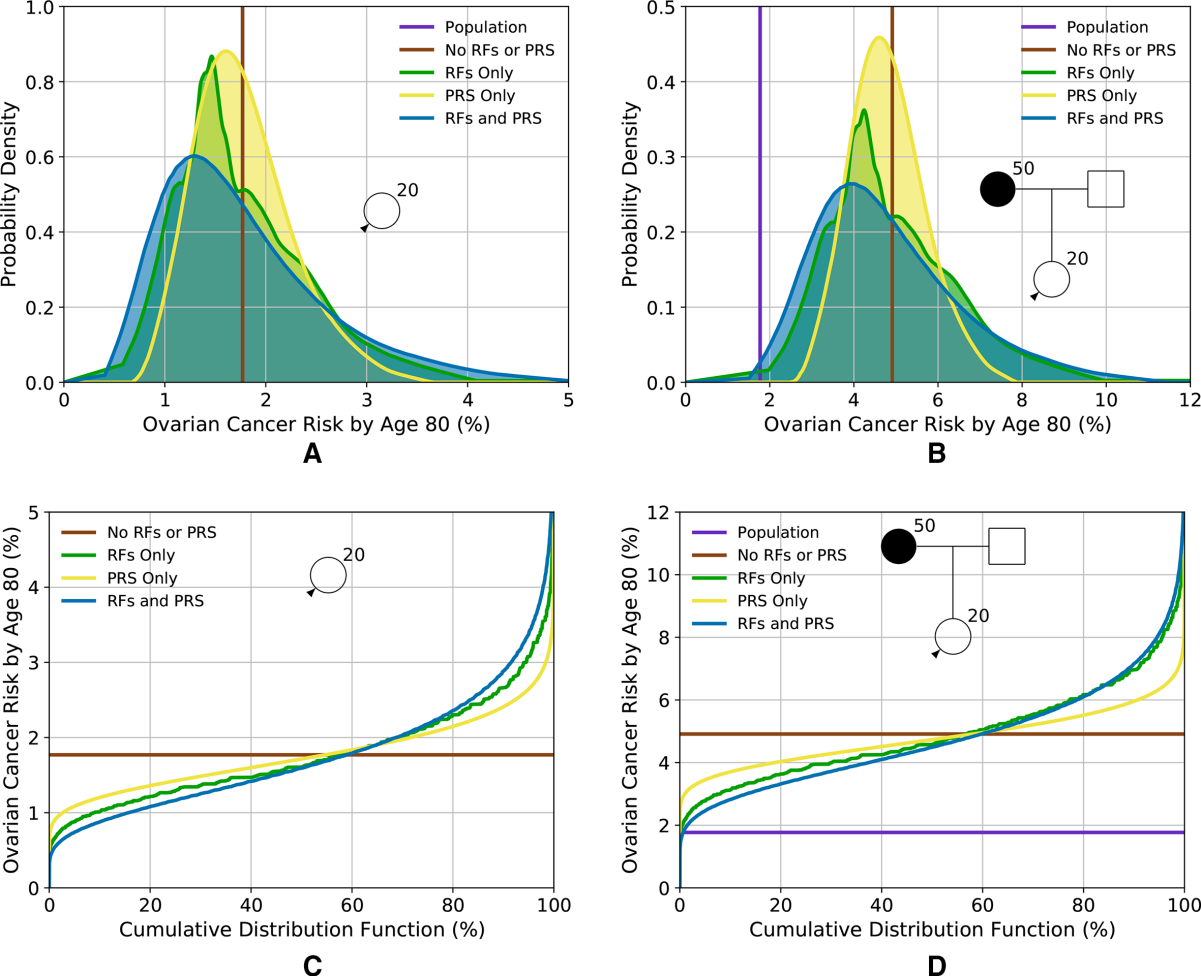
Predicted lifetime (age 20–80 years) EOC risk for a woman untested for PVs based on the different predictors of risk (RFs and PRS). (A, C) Risk for a woman with an unknown family history (equivalent to the distribution of risk in the population); (B, D) risk for a woman with a mother affected at age 50. (A, B) Probability density function against absolute risk; (C, D) absolute risk against cumulative distribution. The vertical line (A) and the horizontal line (C) (labelled ‘no RFs or PRS’) are equivalent to the population risk of EOC. The ‘population’ risk is shown separately in (B, D). Predictions are based on UK EOC population incidences. EOC, epithelial tubo-ovarian cancer; PRS, Polygenic Risk Score; RF, risk factor.

Figure 2 and online supplemental figure S2 show distributions of lifetime risk and risk by age 50, respectively, for women untested for PVs, based on RFs and PRS, for two FH scenarios: (1) unknown FH (ie, equivalent to a woman from the general population); and (2) having a mother diagnosed with EOC at age 50. Table 2 shows the corresponding proportion of women falling into different risk categories. The variation in risk is greatest when including both the RFs and PRS. When considered separately, the distribution is widest for the RFs. Using the RFs and PRS combined, predicted lifetime risks vary from 0.5% for the first percentile to 4.6% for the 99th for a woman with unknown FH and from 1.9% to 10.3% for a woman with an affected mother.

**Table 2:** Percentage of women falling in different risk categories by status of PV in one of the high-risk or intermediate-risk genes included in the model and family history of cancer

PV status	Family history	Risk categories	Lifetime risk			Risk to age 50			Ref fig.
			RF	PRS	RF and PRS	RF	PRS	RF and PRS	
Untested	NA	Population	99.9	100.0	99.4	100.0	100.0	100.0	Figure 2A, C and online supplemental figure S2A, C
		Moderate	0.1	0.0	0.6	0.0	0.0	0.0	
		High	0.0	0.0	0.0	0.0	0.0	0.0	
	M 50	Population	59.1	61.8	61.7	100.0	100.0	100.0	Figure 2B, D and online supplemental figure S2B, D
		Moderate	40.3	38.2	37.1	0.0	0.0	0.0	
		High	0.6	0.0	1.2	0.0	0.0	0.0	
*BRCA1*	NA	Population	0.0	0.0	0.0	0.0	0.0	0.3	Figure 3A and online supplemental figure S3A
		Moderate	0.0	0.0	0.0	1.5	0.1	4.1	
		High	100.0	100.0	100.0	98.5	99.9	95.6	
	M 50	Population	0.0	0.0	0.0	0.0	0.0	0.0	Figure 3B and online supplemental figure S3B
		Moderate	0.0	0.0	0.0	0.0	0.0	0.2	
		High	100.0	100.0	100.0	100.0	100.0	99.8	
*BRCA2*	NA	Population	1.6	0.1	4.7	100.0	100.0	99.9	Figure 3C and online supplemental figure S3C
		Moderate	42.8	37.7	42.3	0.0	0.0	0.1	
		High	55.6	62.2	52.9	0.0	0.0	0.0	
	M 50	Population	0.0	0.0	0.1	95.9	99.9	95.3	Figure 3D and online supplemental figure S3D
		Moderate	1.9	0.1	4.7	4.0	0.1	4.5	
		High	98.1	99.9	95.3	0.0	0.0	0.2	
*RAD51D*	NA	Population	0.6	0.0	2.1	100.0	100.0	99.8	Figure 3E and online supplemental figure S3E
		Moderate	24.5	18.3	30.8	0.0	0.0	0.2	
		High	75.0	81.7	67.1	0.0	0.0	0.0	
	M 50	Population	0.0	0.0	0.0	93.5	99.6	92.6	Figure 3F and online supplemental figure S3F
		Moderate	0.6	0.0	2.0	6.3	0.4	7.0	
		High	99.4	100.0	98.0	0.2	0.0	0.4	
*RAD51C*	NA	Population	1.8	0.1	4.9	99.8	100.0	99.3	Figure 3G and online supplemental figure S3G
		Moderate	44.4	39.3	43.1	0.2	0.0	0.7	
		High	53.7	60.6	52.0	0.0	0.0	0.0	
	M 50	Population	0.0	0.0	0.1	85.8	96.2	85.0	Figure 3H and online supplemental figure S3H
		Moderate	2.0	0.1	5.0	13.6	3.8	13.7	
		High	98.0	99.9	95.0	0.6	0.0	1.3	
*BRIP1*	NA	Population	43.6	34.0	45.7	100.0	100.0	99.7	Figure 3I and online supplemental figure 3I
		Moderate	52.3	65.0	47.4	0.0	0.0	0.3	
		High	4.1	1.0	6.9	0.0	0.0	0.0	
	M 50	Population	2.1	0.1	4.8	93.0	99.3	91.4	Figure 3J and online supplemental figure 3J
		Moderate	43.5	38.9	44.5	6.8	0.7	8.1	
		High	54.5	61.1	50.7	0.3	0.0	0.5	

The ‘population’ risk category is defined as lifetime risk of <5% and risk to age 50 of <3%. ‘Moderate’ risk category is defined as a lifetime risk of 5% or greater but less than 10% and a risk to age 50 of 3% or greater but less than 5%. ‘High’ risk category is defined as a lifetime risk of 10% or greater and a risk to age 50 of 5% or greater. The population lifetime risk is 1.8%, and the population risk to age 50 is 0.27%

EOC, epithelial tubo-ovarian cancer; M 50, mother diagnosed with EOC at age 50; NA, unknown family history; PV, pathogenic variant.

**Figure 3:**
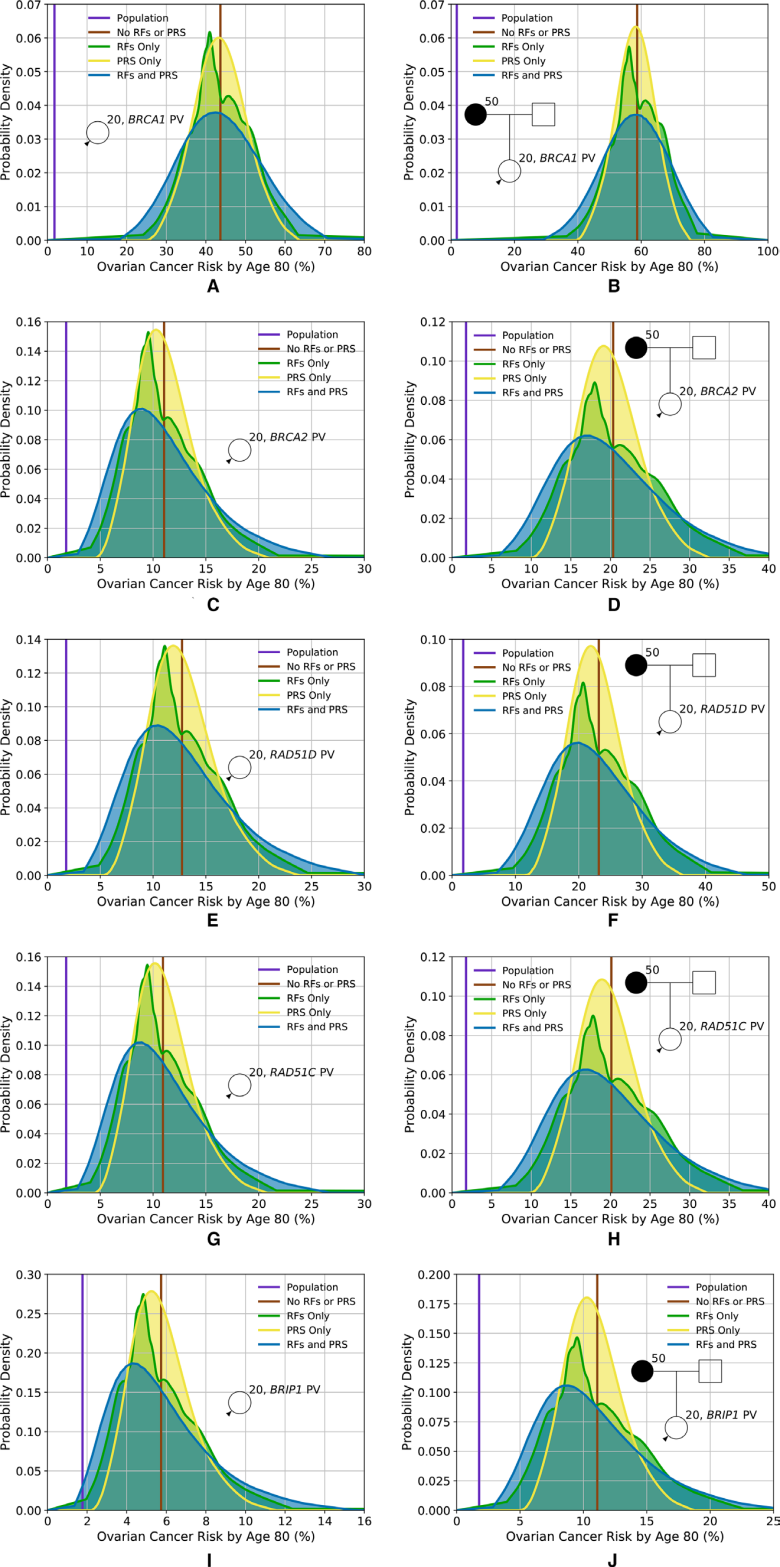
Predicted lifetime EOC risk for a woman who has a PV in one of the high-risk or intermediate-risk genes included in the model, based on the different predictors of risk (RFs and PRS), for two family histories. (A, B) Lifetime risk for a carrier of a PV in *BRCA1*; (C, D) lifetime risk for a carrier of a PV in *BRCA2*; (E, F) lifetime risk for a carrier of a PV in *RAD51D*; (G, H) lifetime risk for a carrier of a PV in *RAD51C*; (I, J) lifetime risk for a carrier of a PV in *BRIP1*. (A, C, E, G, I) Risks for an unknown family history; (B, D, F, H, J) risks for a woman whose mother is diagnosed with EOC at age 50. Predictions based on UK ovarian cancer incidences. EOC, epithelial tubo-ovarian cancer; PRS, Polygenic Risk Score; PV, pathogenic variant; RF, risk factor.

Figure 3 shows the predicted lifetime EOC risk for carriers of PVs in *BRCA1*, *BRCA2*, *RAD51D*, *RAD51C* and *BRIP1* based on RFs and PRS for two FH scenarios. Taking a *RAD51D* PV carrier, for example, based on PV testing and FH alone, the predicted risks are 13% when FH is unknown and 23% when having a mother diagnosed with EOC at age 50. When RFs and the PRS are considered jointly, risks vary from 4% for those at the 1st percentile to 28% for the 99th with unknown FH and from 9% to 43% with an affected mother. Table 1 shows the proportion of women with PVs falling into different risk categories. Based on the combined distribution, 33% of *RAD51D* PV carriers in the population are expected to have a lifetime EOC risk of less than 10%. Similarly, the distributions of risk for *BRIP*1 PV carriers are shown in figure 3I, J and in table 1. Based on the combined RFs and PRS distributions, 46% of *BRIP1* PV carriers in the population are expected to have lifetime risks of less than 5%; 47% to have risks between 5% and 10%, and 7% to have risks of 10% or greater. A *BRIP1* PV carrier with an affected mother, on the basis of FH alone, has a lifetime risk of 11%. However, when the RFs and PRS are considered, 50% of those would be reclassified as having lifetime risks of less than 10%.

Online supplemental figures S4 and S5 show the probability trees describing the reclassification of women as more information (RFs, PRS and testing for PVs in the MGs) is added to the model for a woman with unknown FH and a woman with a mother diagnosed at age 50, respectively, based on the predicted lifetime risks. Online supplemental figures S4A and S5A show the reclassification resulting from adding RFs, MG and PRS sequentially, while online supplemental figures S4B and S5B assume the order RFs, PRS and then MG. Assuming the three risk categories for lifetime risks are <5% and ≥5% but <10% and≥10%, there is significant reclassification as more information is added.

### Model validation

After censoring, 1961 participants with 374 incident cases and 1587 controls met the 5-year risk prediction eligibility criteria. Online supplemental table S5 summarises their characteristics at baseline.

**Figure 4:**
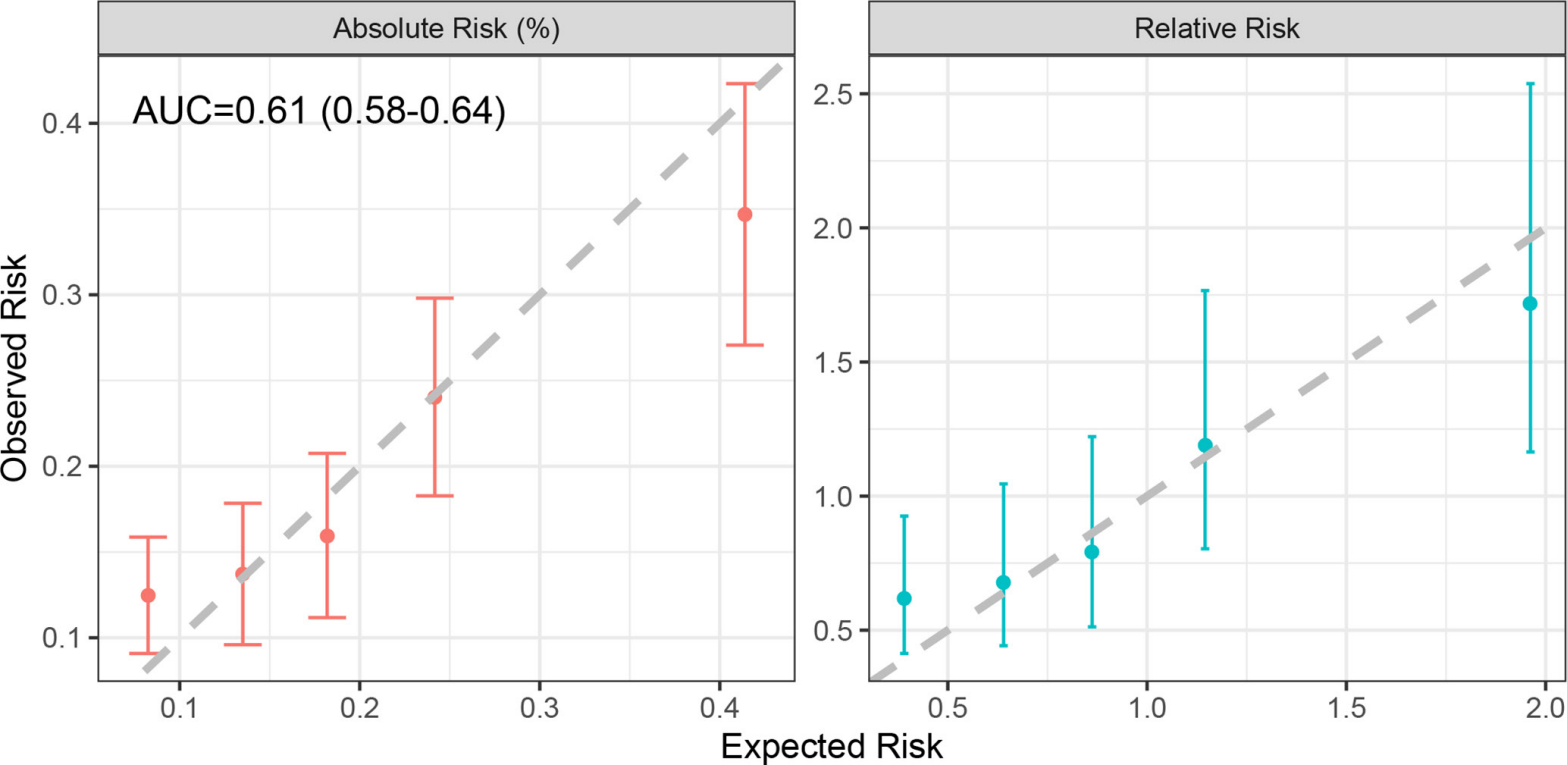
Calibration of the absolute and relative predicted 5-year EOC risks, showing the observed and expected risks by quintile. The bars show the 95% CIs for the observed risks. Relative risks were calculated relative to the overall mean of observed and predicted risks. AUC, area under the receiver operating characteristic curve.

The model considering FH, the 15-variant PRS and a subset of the RFs (but not including testing for PVs in the MGs) demonstrated good calibration in both absolute and relative predicted risk (figure 4). Over the 5-year period, the model predicted 391 EOCs, close to the 374 observed (E/O=1.05, 95% CI: 0.94 to 1.16). The model was well calibrated across the quintiles of predicted risk (HL p=0.08), although there was a suggestion of an underprediction of risk in the lowest quintile (absolute risk E/O=0.66, 95% CI: 0.52 to 0.91; RR E/O=0.63, 95% CI: 0.42 to 0.95). The AUC for assessing discrimination of these model components was 0.61 (95% CI: 0.58 to 0.64).

When looking at individual factors, FH predicted the widest 5 year risk variability (SD=0.0013; range: 0.04% to 4.0%), followed by RFs (SD=0.0010; range: 0.02% to 0.7%) and PRS (SD=0.0009; range: 0.05% to 1.0%, online supplemental figure S6). As expected, their sequential inclusion increased the variability (SD=0.0018; online supplemental figure S6).

## Discussion

The EOC risk prediction model presented here combines the effects of FH, the explicit effects of rare moderate-risk to high-risk PVs in five established EOC susceptibility genes, a 36-variant PRS and other clinical and epidemiological factors (table 1). The model provides a consistent approach for estimating EOC risk on the basis of all known factors and allows for prevention approaches to be targeted at those at highest risk.

The results demonstrate that in the general population (unknown FH), the existing PRS and RF alone identify 0.6% of women who have a lifetime risk of >5% (table 2). On the other hand, for women with FH, 37.1% of women would have a predicted risk between 5% and 10% and 1.2% would have an EOC risk of ≥10% (table 2). The results show that the RFs provide a somewhat greater level of risk stratification than the 36-variant PRS. However, discrimination is greater when both are considered jointly. These results were in line with the observed risk distributions in the validation dataset, but direct comparisons were not possible due to the different variants included in the PRSs and limited RFs in the validation study. The results also show that significant levels of risk recategorisation can occur for carriers of PVs in moderate-risk or high-risk susceptibility genes.

The comprehensive risk model is based on a synthetic approach previously used for BC^30^ and makes several assumptions. In particular, we assumed that the risks associated with known RFs and the PRS combine multiplicatively. We have not assessed this assumption in the present study; however, published studies found no evidence of deviations from the multiplicative model for the combined effect of the RFs and the PRS,^28^ suggesting that this assumption is reasonable. The model assumes that the RFs are also independent of the residual polygenic component that captures the effect of FH. However, for the RFs included, we used estimates from published studies that have adjusted for the other known EOC RFs. The observation that the model was calibrated on the RR scale in the UKCTOCS validation study also suggests that these assumptions are broadly valid.

Similarly, the model assumes that the relative effect-sizes of RFs and the PRS are similar in women carrying PVs in *BRCA1, BRCA2, RAD51C, RAD51D* and *BRIP1* to those without PVs in these genes. Evidence from studies of *BRCA1* and *BRCA2* PV carriers suggests that this assumption is plausible: PRSs for EOC have been shown to be associated with similar RRs in the general population and in *BRCA1* and *BRCA2* PV carriers.^34 38 39^ The current evidence also suggests that known RFs have similar effect sizes in *BRCA1* and *BRCA2* PV carriers as in non-carriers.^40 41^ No studies have so far assessed the joint effects of *RAD51C*, *RAD51D* and *BRIP1* PVs with the PRS, but the observation that FH modifies EOC risk for *RAD51C/D* PV carriers^29^ suggests that similar arguments are likely to apply. Large prospective studies are required to address these questions in more detail. We were not able to validate these assumptions explicitly in UKCTOCS because gene-panel testing data were not available.

Other RFs for EOC that have been reported in the literature include breast feeding^42^ and age at menarche and menopause.^25^ However, the evidence for these RFs is still limited. Our model is flexible enough to allow for additional RFs to be incorporated in the future.

We validated the 5-year predicted risks on the basis of FH, RFs and PRS available in an independent dataset from a prospective trial.^2^ A key strength was that EOC was a primary outcome in UKCTOCS. All cases were reviewed and confirmed by an independent outcome review committee.^2^ The results indicated that absolute and RRs were well calibrated overall and in the top quintiles of predicted risk. However, there was some underprediction of EOC in the bottom quintile. This could be due to differences in the RF distributions in those who volunteer to participate in research (self-selected more healthy individuals^43^) compared with the general population or due to random variations in the effects of the RFs in UKCTOCS compared with other studies. Alternatively, the multiplicative assumption may break down in the lowest-risk category. Further, large prospective cohorts will be required to determine whether the underprediction in the lowest risk category reflects a systematic miscalibration of the model or is due to chance. Although the AUC based on model components in this validation study was modest; it is not surprising given that only a subset of the model predictors were used, and UKCTOCS recruited primarily low-risk women. Inclusion of the optimal PRS,^34^ all RFs and information on PVs in the five genes that account for a large fraction of the EOC FRR are expected to lead to an increase in AUC.

The current validation study has some limitations. The underlying model accounts for FH information on both affected and unaffected family members, but the UKCTOCS recruitment questionnaire did not include information on unaffected family members. Family sizes and ages for unobserved family members were imputed using demographic data. In addition, since information on whether the affected family members were from the paternal or maternal side was absent, we assumed all the affected family members were from the same (maternal) side. This may result in inaccuracies in risk predictions. A further limitation is that UKCTOCS was undertaken to assess screening of low-risk women and therefore is not necessarily representative of a true population cohort, as women with a FH of two or more relatives with EOC or who were known carriers of *BRCA1/2* PVs were not eligible to participate in the randomised controlled trial. Data were not available on the rare moderate-risk and high-risk PVs, and we were only able to assess a PRS with 15 variants, rather than the more informative 36-variant PRS. Therefore, it has not been possible to validate the full model presented here. Future analyses in other cohorts will be required to further validate the full model.

In summary, we have presented a methodological framework for a comprehensive EOC risk prediction model that considers the currently known genetic and epidemiological RFs and explicit FH. The model allows users to obtain consistent, individualised EOC risks. It can also be used to identify target populations for studies to assess novel prevention strategies (such as salpingectomy) or early detection approaches by identifying those at higher risk of developing the disease for enrolment into such studies. Future independent studies should aim to validate the full model, including the full PRS and rare PVs in diverse settings. The model is available via the CanRisk Tool (www.canrisk.org), a user-friendly web tool that allows users to obtain future risks of developing EOC.

## Data Availability

The model is freely available online (www.canrisk.org). For access to UK Collaborative Trial of Ovarian Cancer Screening (UKCTOCS) dataset, which is subject to General Data Protection Regulations rules, please contact the UKCTOCS Biobank coordinator (s.apostolidou@ucl.ac.uk). The data access process is outlined online (http://uklwc.mrcctu.ucl.ac.uk/access-process/).
